# A novel naphthalimide that selectively targets breast cancer via the arylhydrocarbon receptor pathway

**DOI:** 10.1038/s41598-020-70597-8

**Published:** 2020-08-19

**Authors:** J. Gilbert, G. N. De Iuliis, A. McCluskey, J. A. Sakoff

**Affiliations:** 1 Experimental Therapeutics Group, Department of Medical Oncology, Calvary Mater Newcastle Hospital, Edith Street, Waratah, NSW 2298 Australia; 2 Faculty of Science and Information Technology, Priority Research Centre for Reproductive Science, The University of Newcastle, University Drive, Callaghan, NSW 2308 Australia; 3 Chemistry, School of Environmental & Life Sciences, Faculty of Science, The University of Newcastle, University Drive, Callaghan, NSW 2308 Australia

**Keywords:** Cancer, Cell biology, Drug discovery, Oncology

## Abstract

We report that the naphthalimide analogue 2-(2-aminophenyl)-1*H*-benzo[*de*]isoquinoline-1,3(2*H*)-dione (NAP-6) is a highly potent and selective breast cancer targeting molecule. These effects are mediated via the aryl hydrocarbon receptor (AHR) pathway and the subsequent induction of CYP1 metabolising monooxygenases in breast cancer cell line models. Indeed the triple negative breast cancer cell line MDA-MB-468 with a GI_50_ value of 100 nM is greater than 500-fold more sensitive to NAP-6 compared with other tumour derived cell models. Within 1 h exposure of these cells to NAP-6, CYP1A1 expression increases 25-fold, rising to 250-fold by 24 h. A smaller concurrent increase in CYP1A2 and CYP1B1 is also observed. Within 24 h these cells present with DNA damage as evident by enhanced H2AXγ expression, cell cycle checkpoint activation via increased CHK2 expression, S-phase cell cycle arrest and cell death. Specific small molecule inhibitors of the AHR and CYP1 family ameliorate these events. A positive luciferase reporter assay for NAP-6 induced XRE binding further confirms the role of the AHR in this phenomenon. Non-sensitive cell lines fail to show these biological effects. For the first time we identify 2-(2-aminophenyl)-1*H*-benzo[*de*]isoquinoline-1,3(2*H*)-dione as a new AHR ligand that selectively targets breast cancer.

## Introduction

Breast cancer is the most common cancer in women and the incidence is on the rise. Despite advances in hormonal, immunological and targeted therapies, poor response and resistance mechanisms prevail, and metastatic disease is incurable^1^. The aryl hydrocarbon receptor (AHR) pathway has been linked to the induction of breast cancer and to gene activation supporting the progression of this disease^2–9^.

The aryl hydrocarbon receptor (AHR) is a ligand-activated transcription factor and a member of the basic-helix-loop-helix-Per-ARNT-Sim (bHLH-PAS) family. The AHR regulates the transcription of several genes many of which are involved in xenobiotic metabolism. After ligand binding, the complex forms a heterodimer with the AHR nuclear transporter (ARNT), and translocates from the cytosol to the nucleus. The AHR-ARNT heterodimer then interacts with xenobiotic responsive elements (XREs) found on the promoter regions of several target genes. Such xenobiotic genes include the cytochrome P450 metabolising enzymes CYP1A1, CYP1A2 and CYP1B1^10–13^.

Although the AHR is renowned for its ability to metabolise and inactivate environmental toxins, this process can also produce DNA damaging carcinogens that initiate the formation of the cancer phenotype. The role of the AHR in breast cancer is even more sinister than this simple induction process. Indeed, enhanced expression of AHR is associated with malignant progression of the disease^14–16^. Tumour-derived endogenous AHR ligands are known to constitutively activate AHR and drive the expression of CYP1 family members^15, 17^. AHR activates genes involved in inflammation and breast tumour progression^9, 18^. The AHR pathway crosstalks with the estrogen receptor pathway^2, 19, 20^ and also inhibits the function of the DNA damage repair protein BRCA1 via epigenetic silencing^21^. Moreover, suppression of AHR via its repressor protein (AHRR) is associated with metastasis free survival in patients with breast cancer^9^.

**Figure 1:**
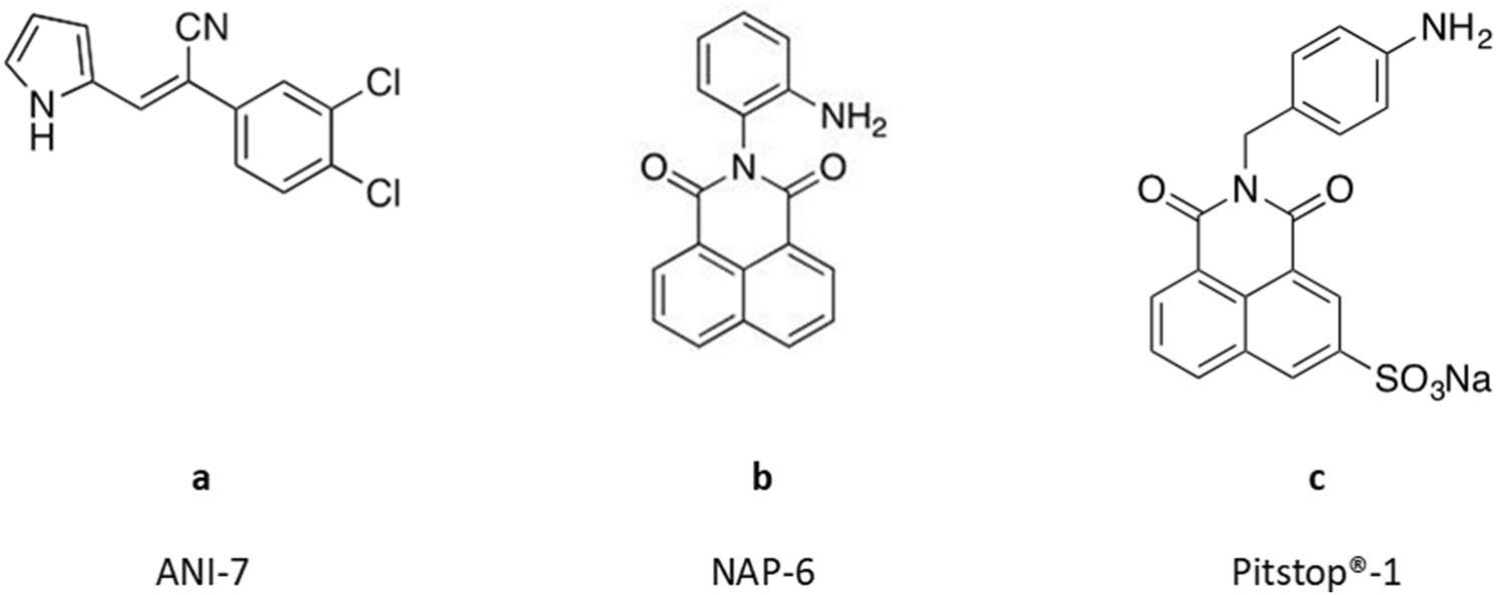
Structure of (**a**) (*Z*)-2-(3,4-dichlorophenyl)-3-(1*H*-pyrrol-2-yl)acrylonitrile (ANI-7); (**b**) 2-(2-aminophenyl)-1*H*-benzo[*de*]isoquinoline-1,3(2*H*)-dione (NAP-6), (**c**) 2-(4-aminobenzyl)-1,3-dioxo-2,3-dihydro-1*H*-benzo[*de*]isoquinoline-5-sulfonic acid sodium salt (Pitstop-1).

We have previously reported the design and synthesis of (*Z*)-2-(3,4-dichlorophenyl)-3-(1*H*-pyrrol-2-yl)acrylonitrile (ANI-7, Fig. 1a), identifying it as a potent and selective inhibitor of cell growth in numerous breast cancer cell lines^22, 23^, while having minimal to no effect on the growth of normal non-tumour derived breast cells or cells derived from other tumour types. We confirmed that this halogenated aryl-hydrocarbon (HAH) mediates its effects via the AHR pathway and the subsequent induction of CYP1 metabolising monooxygenases^23^. Other HAHs that have been investigated in this area include the aminoflavone prodrug (AFP-646) and Phortress (2-(4-amino-3-methylphenyl)-5-fluorobenzothiazole)^24, 25^ both of which have undergone clinical investigation for the treatment of breast cancer. In the present study we show the ability of the alternate AHR ligand class the poly aromatic hydrocarbons (PAH) to selectively target and induce cell death in breast cancer cells. Herein, we report the breast cancer selectivity of a novel naphthalimide class of compound and the role of the AHR pathway in this phenomenon.

## Results

### NAP-6 selectively targets breast cancer cells

We have previously shown that ANI-7 (Fig. 1a), a HAH ligand of the AHR, is a potent (GI_50_ = 0.16 µM) and breast cancer selective (up to 300-fold when compared with other tumour types) growth inhibitor^22, 23^. In the present study, we set out to examine the breast cancer selectivity of a new class of compound, discovered during the phenotypic screening of multiple chemical libraries for dynamin and clathrin inhibitors. The chemical libraries included a number of naphthalimide based compounds including 2-(2-aminophenyl)-1*H*-benzo[*de*]isoquinoline-1,3(2H)-dione (NAP-6) (Fig. 1b) and Pitstop-1 (Fig. 1c). While Pitstop-1 was found to be a potent and non-toxic inhibitor of clathrin^26^, NAP-6 was not an inhibitor of clathrin or dynamin but did produce a unique growth inhibition profile in breast cancer cell lines worthy of further investigation.

**Figure 2:**
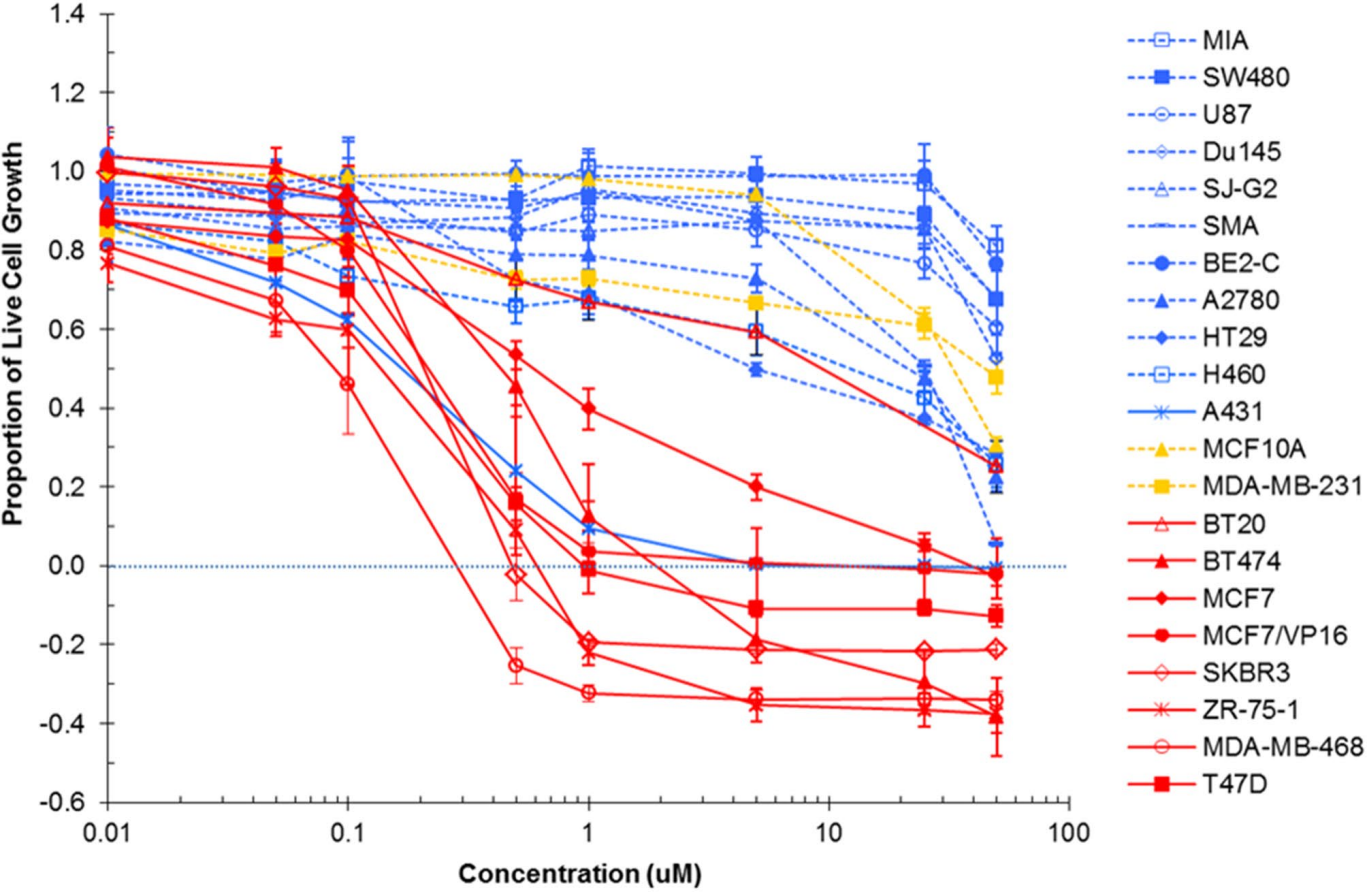
Growth inhibition response (MTT assay) of NAP-6 in various breast (red, orange) and non-breast (blue) derived cell lines after 72 h continuous exposure showing sensitive (solid line) and non-sensitive (dash line) cell populations.

Analysis of the results in Table 1 show the initial growth inhibition screen of NAP-6 and Pitstop-1 in a panel of cancer cell lines derived from a variety of tumour types. At a concentration of 25 µM NAP-6 induced more than 100% growth inhibition in MCF-7 breast cancer cells and a considerably lower effect in the other tumour types. Pitstop-1 was relatively non-toxic at 25 µM; however a preference towards the growth inhibition of MCF-7 cells was noted (28% inhibition). The magnitude of the breast cancer selectivity is more evident when NAP-6 was screened in a broader panel of breast derived cell lines including a non-tumour derived normal MCF10A cell line (Fig. 2). Indeed the GI_50_ values calculated from these dose response curves (Tables 2, 3) show that NAP-6 is active in the MCF-7, BT474, T47D, ZR-75-1, SKBR3 and MDA-MB-468 cells with GI_50_ values of 0.1–0.7 µM. These cells lines are derived from breast cancer tumours with varying receptor status, including ER^+ve^, HER2^+^ and triple negative (TN) classifications. NAP-6 was also active (GI_50_ 0.25 µM) in the MCF-7/VP-16 cell line that overexpresses the ABCC1 drug resistant gene due to progressive exposure to etoposide. Notably, NAP-6 was ineffective in MCF10A normal cells and in MDA-MB-231 cells and only moderately effective in BT20 cells. The only non-breast cancer cell line sensitive to NAP-6 was the A431 vulva cell line (GI_50_ = 0.25 ± 0.12 µM). Collectively, NAP-6 presented with at least a 500-fold selectivity towards the growth inhibition of breast cancer cells (0.1 µM compared with > 50 µM).

**Table 1:** Percentage Growth inhibition of **NAP-6** and Pitstop-1 at 25 µM after 72 h in a broad panel of cancer cell lines using the MTT assay.

	Percentage growth inhibition at 25 µM					
Cell line	MCF-7	A431	H460	HT29	A2780	BE2-C
Tissue of origin	Breast	Skin	Lung	Colon	Ovary	Neural
NAP-6	> 100	67 ± 1	76 ± 2	32 ± 2	18 ± 3	14 ± 3
Pitstop-1	28 ± 5	5 ± 2	7 ± 3	6 ± 3	1 ± 2	9 ± 2
Cell line	SMA	SJ-G2	Du145	SW480	MIA	
Tissue of origin	Brain	Brain	Prostate	Colon	Pancreas	
NAP-6	26 ± 6	16 ± 1	4 ± 5	9 ± 8	10 ± 4	
Pitstop-1	2 ± 1	7 ± 2	< 0	4 ± 1	0 ± 2	

The higher the value the greater the growth inhibition.

**Table 2:** Growth inhibition (GI_50_ µM) of NAP-6 after 72 h in a broad panel of cancer cell lines using the MTT assay.

	GI_50_ (µM)					
Cell line	MCF-7	A431	H460	HT29	A2780	BE2-C
Tissue of origin	Breast	Skin	Lung	Colon	Ovary	Neural
NAP-6	0.70 ± 0.12	0.25 ± 0.12	15 ± 7.5	5.0 ± 0.55	21 ± 4	> 50
Cell line	SMA	SJ-G2	Du145	U87	SW480	MIA
Tissue of origin	Brain	Brain	Prostate	Brain	Colon	Pancreas
NAP-6	25 ± 1	> 50	> 50	> 50	> 50	> 50

The lower the value the greater the growth inhibition.

**Table 3:** Growth inhibition (GI_50_ µM) of NAP-6 after 72 h in a panel of breast cancer cell lines including the non-cancer derived MCF10A cells using the MTT assay.

	GI_50_ (µM)				
Cell line	MCF10A	MDA-MB-231	BT20	MCF-7	BT474
Classification	Normal^a^	TN^b^	TN^b^	ER+^c^	ER+^c^
NAP-6	31 ± 1.5	35 ± 3	14 ± 1.5	0.70 ± 0.12	0.43 ± 0.07
Cell line	T47D	ZR-75-1	MCF-7/VP16	SKBR3	MDA-MB-468
Classification	ER+^c^	ER+^c^	ER+^c,e^	HER2+^d^	TN^b^
NAP-6	0.18 ± 0.02	0.12 ± 0.03	0.25 ± 0.06	0.22 ± 0.02	0.10 ± 0.02

^a^Normal breast cell line.

^b^Triple negative (TN) for ER, PR and HER2.

^c^Estrogen receptor positive (ER+).

^d^ER negative HER2 positive.

^e^Etoposide resistant MCF-7 clone.

### NAP-6 induces cell cycle arrest, checkpoint activation and DNA damage

**Figure 3:**
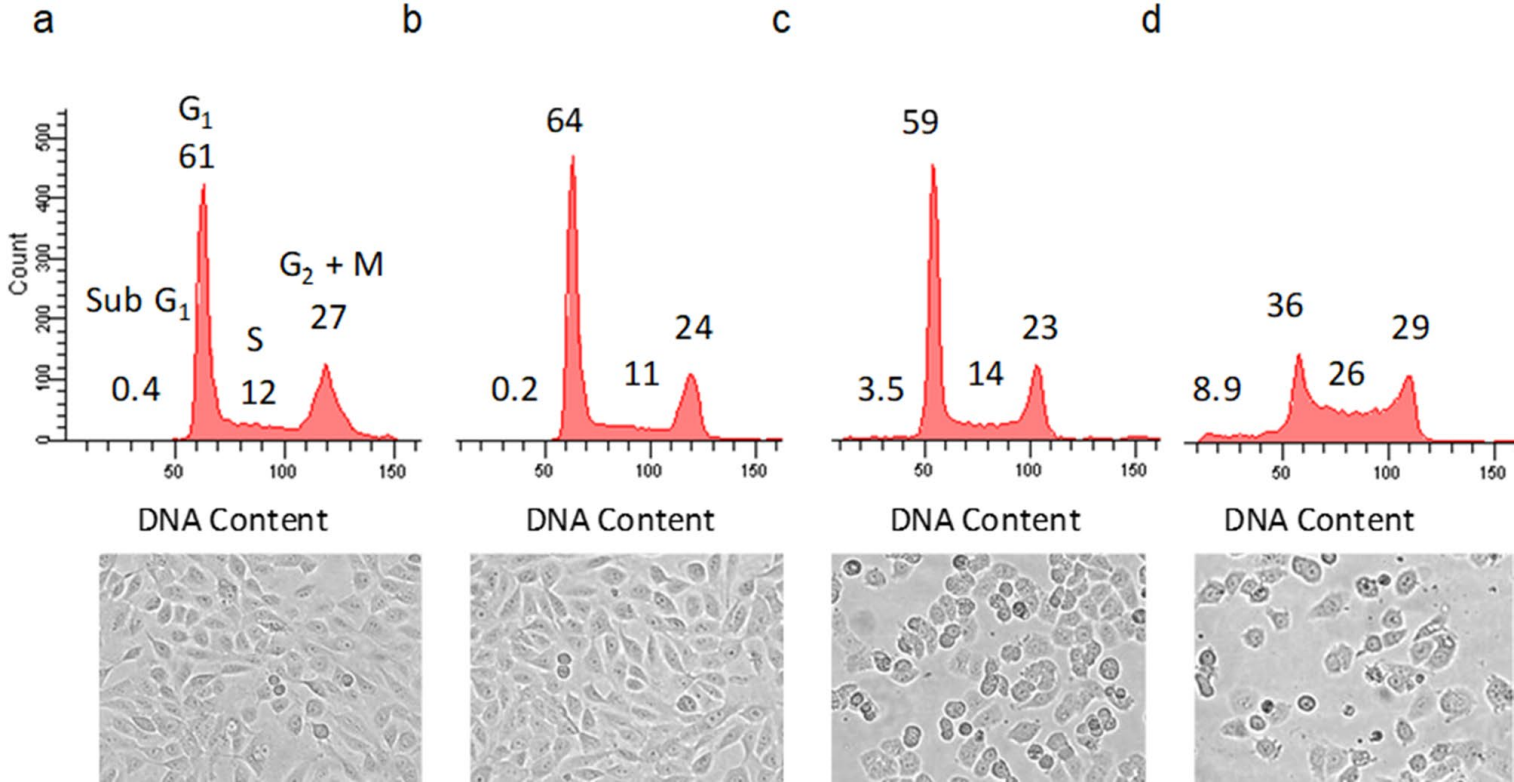
Cell cycle analysis (percentage distribution) and corresponding morphological assessment of MCF10A (**a**,**b**) and MDA-MB-468 (**c**,**d**) cells treated with (**b**,**d**) or without (**a**,**c**) NAP-6 (1 µM) for 24 h.

**Figure 4:**
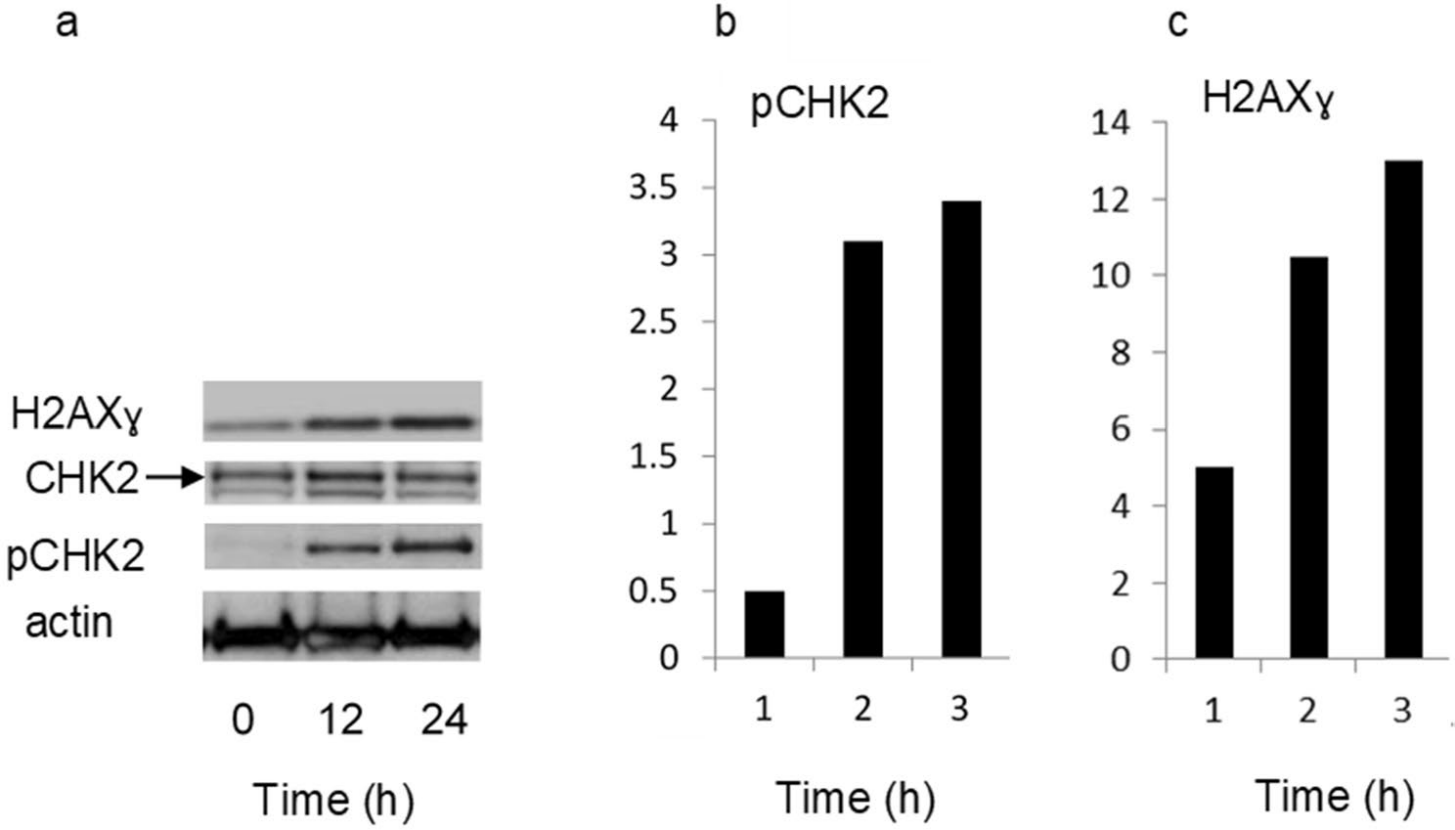
MDA-MB-468 cells were treated with NAP-6 (1.0 μM) for 0, 12 and 24 h, and examined for checkpoint activation (CHK2, pCHK2) and DNA damage (H2AXɣ) by Western blotting. Full length uncropped gels are shown in supplementary information. The relative optical density normalised to actin content is also shown. Data was replicated on two separate occasions, with one representative set shown.

To further investigate the mechanism-of-action of NAP-6 we decided to examine the cell cycle events induced in the most sensitive cell line MDA-MB-468. Cell cycle analysis (Fig. 3) and morphological assessment of NAP-6 confirmed the negligible effect of NAP-6 (1 µM) on the growth of normal breast MCF10A cells within 24 h (Fig. 3a,b), while NAP-6 induced substantial S-phase and G_2_ + M phase cell cycle arrest in MDA-MB-468 cells (Fig. 3c,d). Western blot gel analysis (Fig. 4a) showed the induction of cell cycle checkpoint activation within 12 h of treatment with NAP-6 (1 µM) in MDA-MB-468 cells, via a substantial increase (sixfold) in the phosphorylation of CHK2 (Fig. 4b). Concurrently, NAP-6 (1 µM) induced a substantial increase in H2AXɣ (more than twofold) in MDA-MB-468 cells under the same conditions, indicative of DNA double strand damage (Fig. 4c). The propensity of NAP-6 to induce S-phase cell cycle arrest and selectively target breast cancer cells is typical of compounds that target the AHR pathway^23, 27^.

### Inhibition of the AHR pathway ameliorates the effects of NAP-6

**Figure 5:**
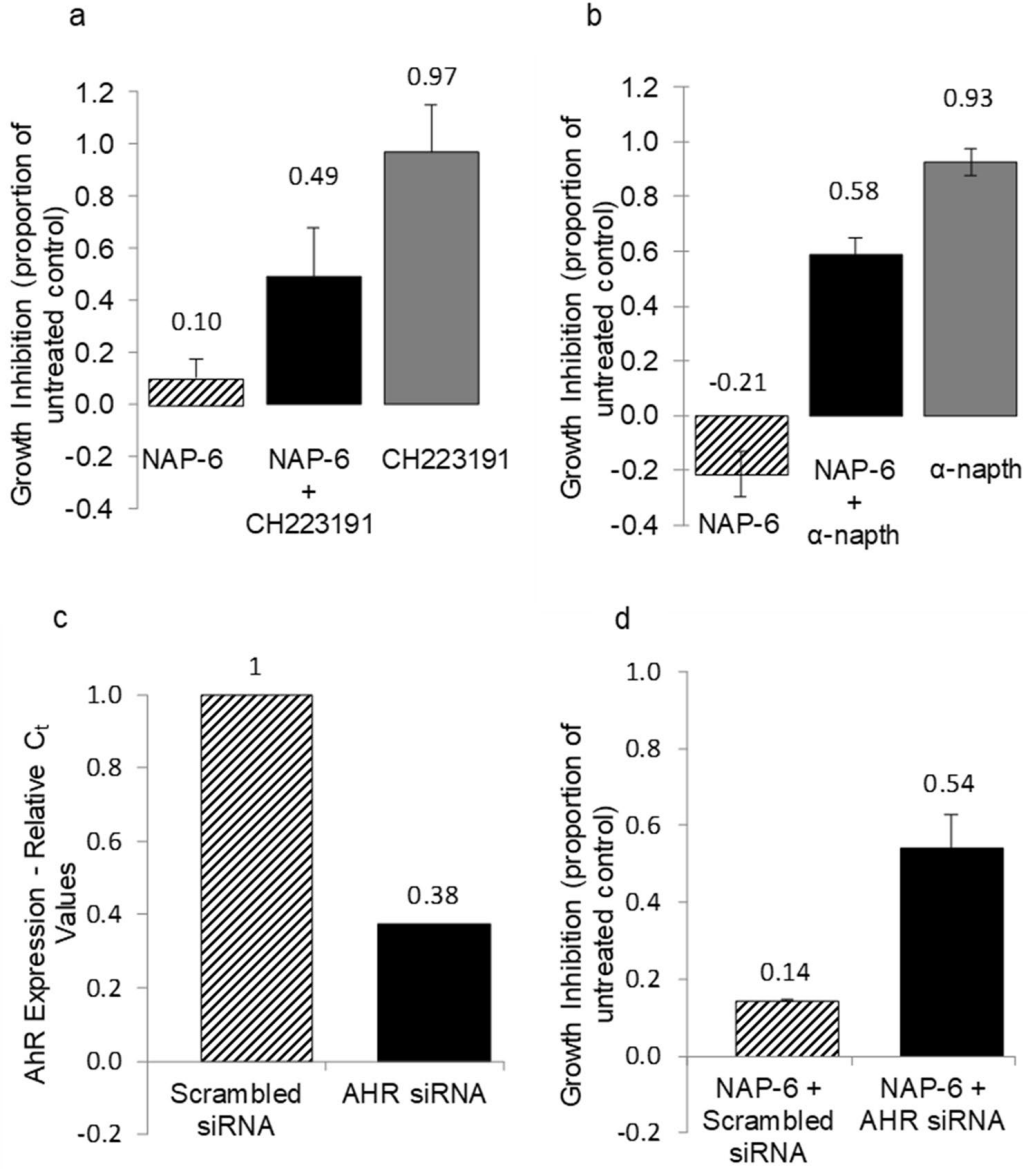
Growth inhibition response (MTT assay) in MDA-MB-468 cells after 72 h of (**a**) NAP-6 (0.1 μM) in the presence and absence of the AHR antagonist CH223191 (5 μM) and (**b**) NAP-6 (0.5 μM) in the presence and absence of the CYP1 inhibitor α-naphthoflavone (αNF) (10 μM). Each data point is the mean ± SEM of three replicates. (**c**) Fold change in expression of AHR in MDA-MB-468 cells in the presence (AHR siRNA) or absence (scrambled siRNA) of AHR knockdown. (**d**) Growth inhibition response in MDA-MB-468 cells after 48 h of NAP-6 (0.5 μM) in the presence (AHR siRNA) or absence (scrambled siRNA) of AHR siRNA. Each data point is the mean ± SEM of two replicate experiments.

To confirm the role of NAP-6 in activating the AHR pathway we examined the effect of the AHR antagonist, CH223191 (5 µM)^28^, on the ability of NAP-6 to induce growth inhibition. The results in Fig. 5a show a substantial increase in survival of MDA-MB-468 cells in response to NAP-6 (0.1 μM) from 10 to 50% in the presence of CH223191 (5 µM). Since the AHR pathway is also known to induce the expression of CYP1 cytochrome P450 enzymes the effect of the specific CYP1 inhibitor α-naphthoflavone was also examined. Indeed, α-naphthoflavone (10 µM) ameliorated the growth inhibitory effects of NAP-6 (0.5 µM) from total growth inhibition to 58% survival (Fig. 5b). Furthermore, siRNA knockdown of AHR expression by 60% (Fig. 5c) enhanced the survival of NAP-6 (0.5 µM) treated cells from 14 to 54% (Fig. 5d). For assay optimisation the highest concentration of inhibitor that induced the least effect on cell growth was used; similarly the concentration of NAP-6 was optimised by titrating NAP-6 with each inhibitor and observing the maximal impact on growth amelioration. Collectively, these observations confirm the role of the AHR and CYPs in mediating the effects of NAP-6 in sensitive breast cancer cell line models. Supplementary Fig. S2 shows that NAP-6 with or without CH223191 or α-naphthoflavone had no effect on cell growth in the normal MCF10A cells.

### NAP-6 activates XRE activity and expression of CYP1 family members

**Figure 6:**
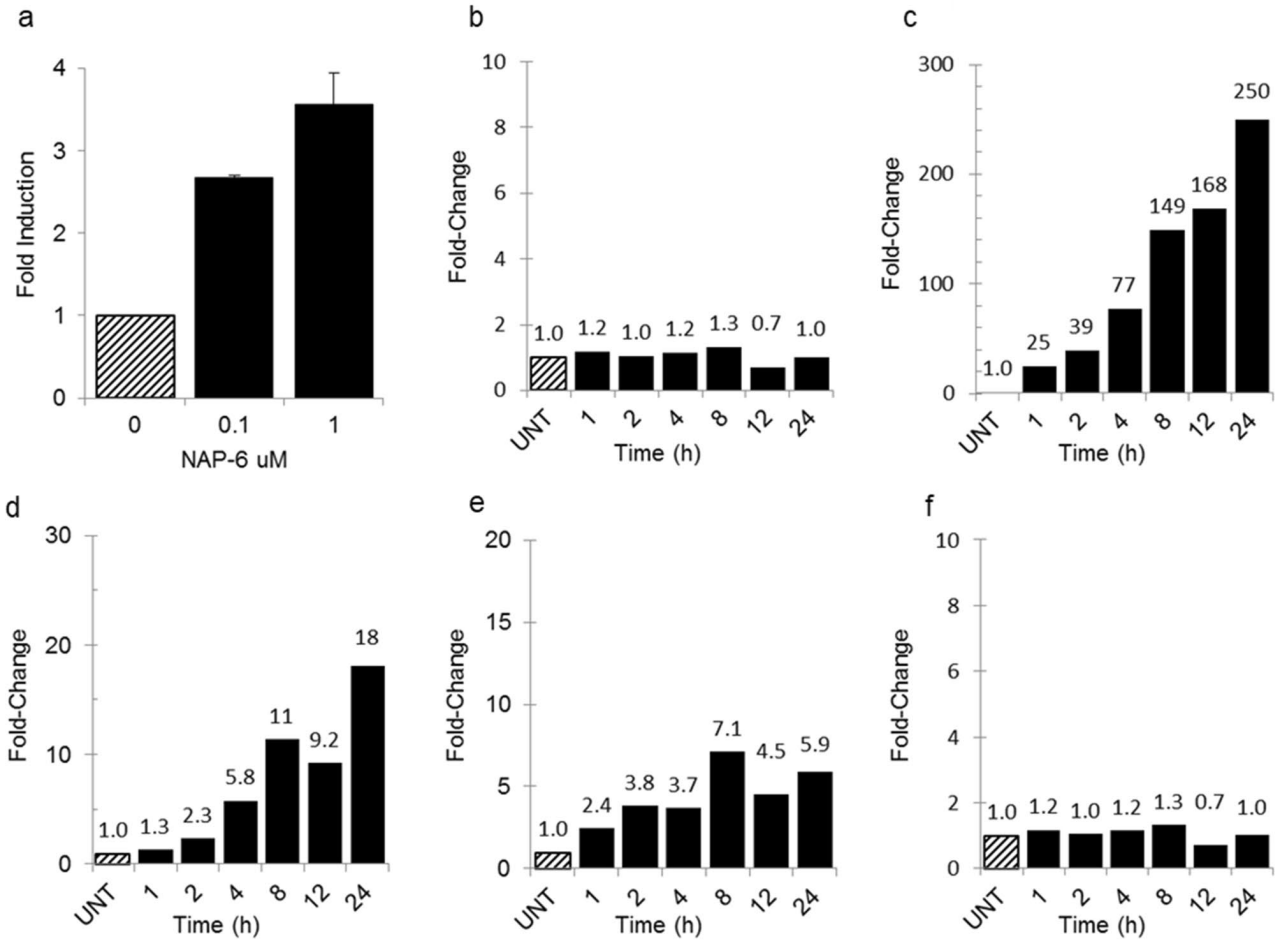
(**a**) Induction of XRE activity using a reporter assay in MDA-MB-468 cells after 6 h of NAP-6 (0.1 and 1 μM) treatment. Each data point is the mean ± SEM of two replicate experiments. (**b**–**f**) Change in gene expression (q PCR) in MDA-MB-468 cells of (**b**) AHR, (**c**) CYP1A1, (**d**) CYP1A2, (**e**) CYP1B1 and (**f**) SULT1A1 after 1–24 h treatment of NAP-6 (1 μM) compared with untreated control cells (UNT).

An XRE reporter assay was exploited to determine the ability of NAP-6 to induce binding of the AHR with the XRE promotor. The NAP-6 sensitive cell line MDA-MB-468 was transfected with an XRE reporter plasmid, together with control reporter plasmids. Treatment with NAP-6 at concentrations of 0.1 and 1.0 µM substantially induced promotor activity by up to 3.5-fold within 6 h (Fig. 6a), confirming XRE activation. Expression analysis of AHR in response to treatment with NAP-6 showed no changes within 24 h (Fig. 6b) however; substantial change in the expression of CYPs was noted. Specifically, NAP-6 induced a 25-fold increase in CYP1A1 expression within 1 h of treatment, rising to a 250-fold increase by 24 h (Fig. 6c). Concurrent, albeit smaller, increases in CYP1A2 and CYP1B1, up to 18-fold and sixfold respectively were observed under the same conditions (Fig. 6d,e). Interestingly, no changes in the expression of the phase 2 metabolising enzyme SULT1A1 was observed following treatment (Fig. 6f).

**Figure 7:**
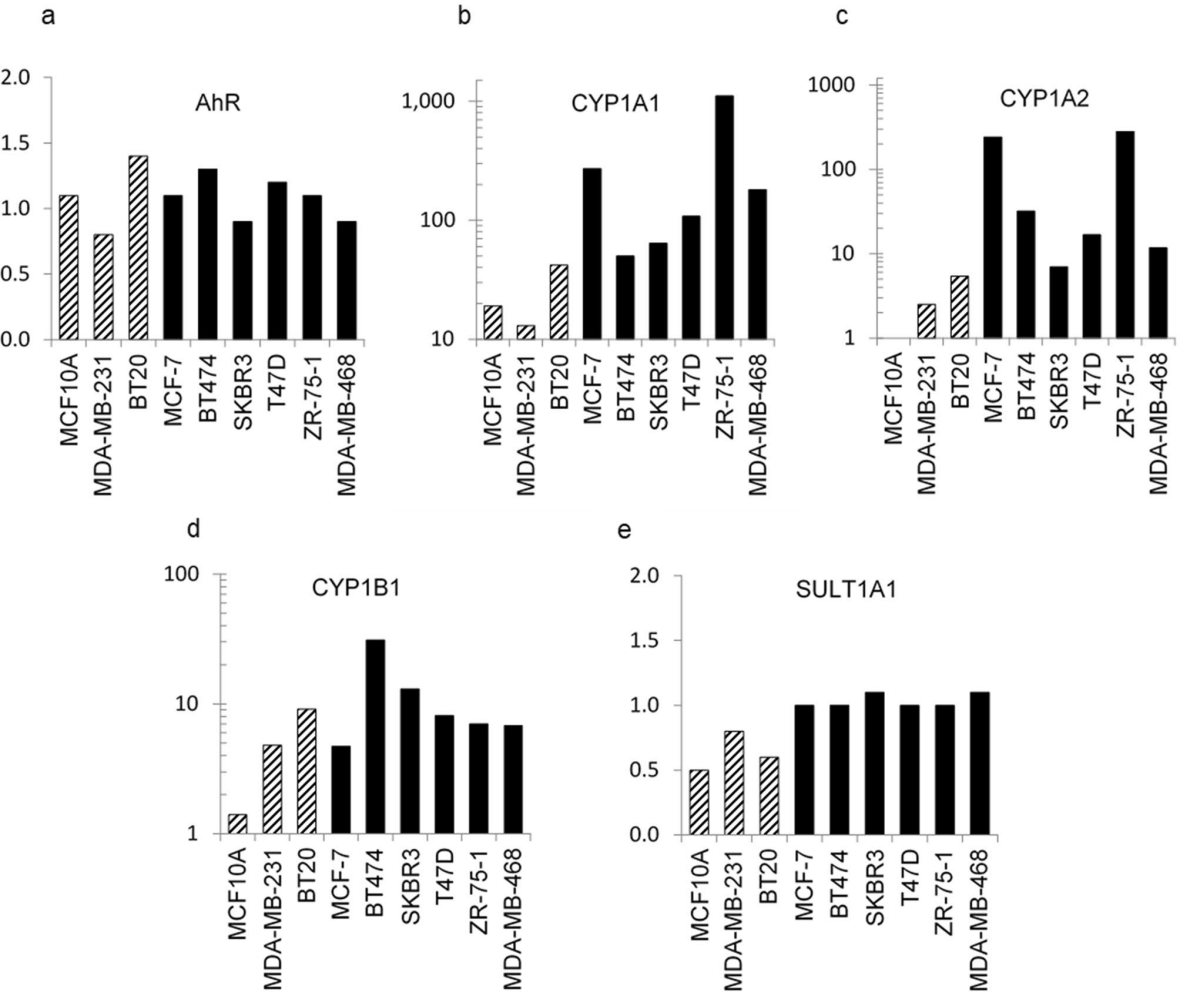
Fold change in gene expression (qPCR) of (**a**) AHR, (**b**) CYP1A1, (**c**) CYP1A2, (**d**) CYP1B1 and (**e**) SULT1A1 compared with untreated cells in MCF10A, MDA-MB-231, BT20, MCF-7, BT474, SKBR3, T47D, ZR-75–1, and MDA-MB-468 cells after 4 h treatment of NAP-6 at 10 × the GI_50_ for each cell line (see Table 2).

A similar profile of enhanced CYP expression (fold-change after 4 h of treatment) was observed in the other NAP-6 sensitive MCF-7, BT474, SKBR3, T47D, ZR-75-1 breast cancer cell lines, while the non-sensitive MCF10A and MDA-MB-231 failed to induce this response (Fig. 7). Collectively the ability of the sensitive cell lines to respond to NAP-6 primarily correlated with the induction of CYP1A1 and CYP1A2 expression.

### Potential off-targets of NAP-6

In order to further characterise the activity of NAP-6 we also evaluated its ability to inhibit the ER receptor, aromatase activity and enzymatic activity of a broad panel of protein kinases. An eight point dose response curve of NAP-6 failed to show ER binding even at the highest concentration of 2 µM. In contrast, tamoxifen and 4-hydroxytamoxifen substantially bound to the receptor with IC_50_ values of 0.012 ± 0.009 µM and 0.0017 ± 0.007 µM, respectively (Table 4). NAP-6 also failed to inhibit aromatase activity at concentrations up to 100 µM while anastrozole showed substantial inhibition with an IC_50_ value of 0.12 ± 0.07 µM (Table 4). In a broad panel of kinase enzymes including tyrosine kinase receptors, lipid kinases, or those specific to the PI3K/mTOR or MAP Kinase pathway, NAP-6 at a concentration of 10 µM did not substantially alter kinase activity (Table 5).

**Table 4:** In vitro ER and aromatase inhibition (IC_50_ µM) assay in the presence of NAP-6, Tamoxifen, hydroxytamoxifen and anastrozole.

	ER inhibition	Aromatase inhibition
	(IC_50_ µM)	(IC_50_ µM)
NAP-6	> 2	> 100
Tamoxifen	0.012 ± 0.009^a^	nd^b^
4-Hydroxytamoxifen	0.0017 ± 0.0007^a^	nd
Anastrazole	nd	0.12 ± 0.07^a^

Data represents the mean ± SEM of three replicate experiments.

^a^From Gilbert et al.^23^.

^b^Not determined.

**Table 5:** Percentage kinase activity in the presence of 10 µM NAP-6.

Kinase classifications			
**Receptors**		**PI3K/mTOR pathway**	
EGFR	105 ± 4.7^a^	PDK1/PDPK1	100 ± 1.6^a^
ERBB2/HER2	91 ± 0.8^a^	SGK1	97 ± 0.6^a^
ERBB4/HER4	113 ± 0.7^a^	SGK2	102 ± 1.5^a^
IGF1R	85 ± 1.4^b^	SGK3/SGKL	106 ± 0.2^a^
PDGFRa	115 ± 5.6^a^	AKT1	92 ± 1.5^a^
PDGFRb	119 ± 1.9^a^	AKT2	90 ± 3.3^a^
**Lipid kinases**		AKT3	105 ± 0.1^a^
Choline kinase alpha	97 ± 2.7^b^	COT1/MAP3K8	92 ± 2.9^a^
Choline kinase beta	99 ± 2.6^b^	GSK3a	94 ± 1.3^a^
DGK beta	70 ± 2.2^b^	GSK3b	98 ± 2.1^a^
DGK gamma	39 ± 3.6^b^	mTOR/FRAP1	102 ± 0.4^a^
DGK zeta	94 ± 12^b^	ROCK1	100 ± 1.5^a^
PI3 kinase alpha	86 ± 19^a^	ROCK2	99 ± 2.5^a^
PI3 kinase beta	107 ± 5.9^a^	P70S6K	89 ± 7.7^b^
PI3 kinase delta	87 ± 3.6^a^	**MAP kinase pathway**	
PI3 kinase gamma	92 ± 16^a^	RAF1	104 ± 0.5^a^
PI4K2A	97 ± 5.2^b^	ARAF	102 ± 0.6^a^
PIP5K2A	92 ± 4.7^b^	BRAF	105 ± 1.7^a^
Sphingosine kinase 1	77 ± 1.0^b^	MEK1	108 ± 0.1^a^
Sphingosine kinase 2	89 ± 8.7^b^	MEK2	97 ± 1.0^a^
**Others**		ERK1	101 ± 3.6^a^
BRK	90 ± 2.4^a^	ERK2/MAPK1	100 ± 0.8^a^
PKBa	106 ± 1.3^b^	c-Src	88 ± 1.5^a^
PKD1	87 ± 6.1^b^		
PKBb	89 ± 4.0^b^		

The data represent the mean ± SEM of duplicate experiments.

^a^Reaction Biology Corporation USA.

^b^The International Centre for Kinase Profiling University of Dundee UK.

## Discussion and conclusion

We report that the naphthalimide analogue 2-(2-aminophenyl)-1*H*-benzo[*de*]isoquinoline-1,3(2*H*)-dione (NAP-6) is a potent (nM) and selective (more than 500-fold) inhibitor of cell growth in breast cancer cell line models. Importantly, the cell lines sensitive to treatment are derived from breast cancer molecular subtypes of luminal A (MCF-7, T47D, ZR-75-1), luminal B (BT474), basal (MDA-MB-468, BT20) and HER2 (SKBR3) with varying receptor status for ER, PR, and HER2. Also included is the MCF-7/VP16 cell line which presents with a drug resistant phenotype (MCF-7/VP16) and overexpresses the p-glycoprotein drug transporter ABCC1.

Notably, all ER positive lines including MCF-7, BT474, T47D, ZR-75-1 and MCF7/VP16 were sensitive to growth inhibition of NAP-6. Similar sensitivity was also shown for two ER negative cell lines, i.e. SKBR3 and MDA-MB-468 cells, with the latter the most sensitive. This profile of sensitivity to AHR ligands has previously been described by us and others^23, 29–31^. The non-sensitive MDA-MB-231 cell line is triple negative for receptor status and represents a basal subtype of breast cancer with amplifying mutations in KRas and BRaf activity^32^. Such mutations are rarely found in breast cancer^33^. The resistance of MDA-MB-231 cells to AHR activation has been observed with other ligands including aminoflavone and ANI-7^19, 23, 34^. The only non-breast cancer cell line that showed appreciable sensitivity to NAP-6 was the A431 skin-vulva cell line, an ER positive cell line overexpressing the EGFR growth receptor^35^. These observations in cell line sensitivity mirror those observed for the HAH AHR ligand ANI-7 and aminoflavone previously reported by us, albeit of a differing chemical class^23^.

The naphthalimide core structure of NAP-6 is observed in other chemotherapy treatments including amonafide, and mitonafide^36^. Amonafide, a topoisomerase II inhibitor was assessed in a Phase III clinical trial in combination with cytarabine for treatment of secondary acute myeloid leukemia. This disease is characterised by overexpression of drug efflux mechanisms including the gp170 protein, of which amonafide is not a substrate. Unfortunately this combination was no better than standard treatment^36^. Sankara et al.^37^, designed various naphthalimide benzothiazole/cinnamide analogues, all of which showed DNA intercalation, topoisomerase inhibition and low µM cytotoxicity using the MTT assay; however, none showed breast cancer selectivity in MCF-7 cells. Li et al.^17^ designed various naphthalimide-pyrazolyl derivatives that again showed DNA intercalation properties; the most potent of these showed a slight selectively towards MCF-7 cells (MTT assay 0.7 µM) compared with HeLa (3 µM) and A549 (5 µM) cells. Further to these observations a comprehensive review of more than 750 1,8-naphthalimide analogues was conducted in 2018, while many were effective at inhibiting the growth of cancer cell lines, none presented with the breast selectivity observed in the present study nor was the AHR pathway noted in their mode of action^38^.

Using standard cell biology methods we show that NAP-6 binds to the AHR, induces translocation to the nucleus, activates the XRE (Fig. 6a), induces CYP1 activity (Fig. 6c–e), culminating in cell cycle arrest (Fig. 3), checkpoint activation (Fig. 4), DNA damage (Fig. 4) and cell death (Fig. 2). Of note, is the substantial induction of CYP1 expression within 1 h following treatment, with CYP1A1 dominating the effect (Fig. 6c). The amelioration of this effect by AHR antagonists, AHR siRNA and CYP inhibition (Fig. 5) further supports the role of the AHR pathway. The enhanced expression of CYP1A1, CYP1A2, and CYP1B1 in the other NAP-6 sensitive cells lines (Fig. 7) confirms that this phenomenon is not specific for the MDA-MB-468 cells but rather the mode-of-action across all sensitive populations.

In previous studies we have examined the inherent expression of the AHR family members in the current panel of cell lines^23^. Comparisons show that the ability of NAP-6 to activate the AHR is not dependent upon the inherent expression of the pathway members (AHR, ARNT and CYP1), underscoring their inducible nature rather than constitutive activity^23^. However, comparison with the inherent expression of SULT1A1 did predict for NAP-6 sensitivity and its expression was not altered following treatment (Fig. 6). The role of the phase 2 metabolising enzyme SULT1A1 has been noted in the breast cancer selectivity of ANI-7 and aminoflavone^23, 27^. Moreover, the transfection of SULT1A1 into MDA-MB-231 aminoflavone-resistant cells restored sensitivity^27^, highlighting the multiple steps of drug metabolism. Structurally, aryl amine groups are metabolised by CYPs to form *N*-hydroxyl metabolites that are then substrates for bioactivation by sulphur transferase (SULT1A1). *N*-sulfoxy groups are further converted to active nitrenium ions, which form DNA adducts and induce cell death^27^. The presence of the DNA damage marker H2AXɣ and cell cycle checkpoint activation suggest that NAP-6 is metabolised to a DNA interacting compound; although the identity of this metabolite is unknown.

For the first time we have identified the naphthalimide analogue 2-(2-aminophenyl)-1*H*-benzo[*de*]isoquinoline-1,3(2*H*)-dione (NAP-6) as a potent and selective inhibitor of breast cancer cell growth. This is the first 1,8-naphthalimide analogue to show this effect. This study builds upon our knowledge of selectively targeting breast cancers and significantly adds to the development of molecules exploiting the AHR pathway.

## Materials and methods

### Cell lines

All test agents were prepared as stock solutions (20 mM) in dimethyl sulfoxide (DMSO) and stored at − 20 °C. CH223191 and α-naphthoflavone were purchased from Sigma (Australia). Cell lines used in the study included MCF-7, MDA-MB-468, T47D, ZR-75-1, SKBR3, BT474, BT20, MDA-MB-231, MCF7/VP16 (breast carcinoma); HT29, SW480 (colorectal carcinoma); U87, SJ-G2, SMA (glioblastoma); A2780 (ovarian carcinoma); H460 (lung carcinoma); A431 (skin carcinoma); Du145 (prostate carcinoma); BE2-C (neuroblastoma); and MiaPaCa-2 (pancreatic carcinoma) together with the one non-tumour derived normal breast cell line (MCF10A). All cell lines were incubated in a humidified atmosphere 5% CO_2_ at 37 °C. The cancer cell lines MCF7, MCF7/VP16, MDA-MB-231, HT29, SW480, U87, SJ-G2, SMA, A2780, H460, A431, DU145, BE2-C and MIAPaCa2 were maintained in Dulbecco’s modified Eagle’s medium (DMEM; Sigma, Australia) supplemented with foetal bovine serum (10%), sodium pyruvate (10 mM), penicillin (100 IU mL^−1^), streptomycin (100 µg mL^−1^), and l-glutamine (2 mM). The cancer cell lines MDA-MB-468, T47D, ZR-75–1, SKBR3 and BT474 were maintained in RPMI-1640 (Sigma, Australia) supplemented with foetal bovine serum (10%), sodium pyruvate (10 mM), penicillin (100 IU mL^−1^), streptomycin (100 µg mL^−1^), l-glutamine (2 mM) and HEPES (10 mM). The non-cancer MCF10A cell line was maintained in DMEM:F12 (1:1) cell culture media, 5% heat inactivated horse serum, supplemented with penicillin (50 IU mL^−1^), streptomycin (50 µg mL^−1^), HEPES (20 mM), l-glutamine (2 mM), epidermal growth factor (20 ng mL^−1^), hydrocortisone (500 ng mL^−1^), cholera toxin (100 ng mL^−1^), and insulin (10 mg mL^−1^)^23^.

### Growth inhibition

Growth inhibition was determined by plating cells in duplicate in medium (100 µL) at a density of 2,500–4,000 cells per well in 96-well plates. On day 0 (24 h after plating), when the cells are in logarithmic growth, medium (100 µL) with or without the test agent was added to each well. After 72 h drug exposure, growth inhibitory effects were evaluated using the MTT (3-(4,5-dimethyltiazol-2-yl)-2,5-diphenyltetrazolium bromide) assay and absorbance read at 540 nm^39^. The percentage growth inhibition was calculated at a fixed concentration of 25 µM (Table 1). The GI_50_ value was calculated from an eight-point dose–response curve as shown in Fig. 2 using MS Excel software. Each data point is the mean ± the standard error of the mean (SEM) calculated from 4–5 replicates which were performed on separate occasions and separate cell line passages. The GI_50_ value represents the drug concentration at which cell growth was inhibited by 50% based on the difference between the optical density values on day 0 and those at the end of drug exposure (Tables 2, 3).

### Cell cycle analysis

Cells in logarithmic growth were transferred to 6 well plates at a density of 2 × 10^5^–2.5 × 10^5^ cells/well. On day 0 (24 h after plating), the cells were treated with or without NAP-6. The cells were harvested 24 h after drug treatment and washed twice in phosphate buffered saline (PBS), fixed in 70% ethanol and stored overnight at − 20 °C. The cell pellet was incubated in 600 µL of PBS containing propidium iodide (40 µg mL^−1^) and RNase (200 µg mL^−1^) for at least 30 min at room temperature. The samples (1.5 × 10^4^ events) were analysed for fluorescence (FL2 detector, filter 575/30 nm band pass) using a FACScan (Becton Dickinson)^23^. Cell cycle distribution was assessed using Cell Quest software. Experiments were each performed on three separate occasions. Values are the percentage distribution for each phase of the cell cycle (Fig. 3).

### Morphological assessment

Live cells were examined for morphological alterations after 24 h exposure with and without 1 µM NAP-6, using phase contrast microscopy (Olympus CKX41 inverted microscope  × 100 magnification)^23^ (Fig. 3).

### Western blotting

Cells (3 × 10^5^) were plated in 6 well plates in DMEM media containing test agent. At the indicated times the cells were harvested and protein content determined (Lowry Modified/Biorad Protein Assay). Equal aliquots (20 µg) of total protein from whole cell lysates were fractionated on a 10% denaturing sodium dodecyl sulfate (SDS) polyacrylamide gel and transferred to polyvinylidine difluoride membranes. Nonspecific interactions were blocked with 5% nonfat milk/0.05% Tween 20. Proteins were identified using rabbit monoclonal antibodies against H2AXγ, and pCHK2 (Cell signaling) and mouse monoclonal antibody CHK2. Membrane-bound antibodies were detected using goat anti-rabbit and anti-mouse secondary antibodies (Abcam) and Clarity Western ECL (Bio-Rad)^23^. Full length gels are shown in Supplementary Fig. S1.

### AHR knockdown

Transient knockdown of AHR in MDA-MB-468 cells was performed through transfection of small interfering RNAs (siRNA) targeting AHR (Qiagen) and the AllStars Negative Control nonsilencing siRNA (Qiagen). The AHR siRNA contained four siRNAs for the AHR target (FlexiTube GeneSolution GS196). Cells were transfected with Lipofectamine 3000 (Invitrogen) according to the manufacturer’s instructions. Briefly, 8 × 10^3^ MDA-MB-468 cells were plated into each well of a 96-well plate and allowed to adhere for 24 h. Opti-MEM media (Invitrogen) containing 0.3 mL of Lipofectamine 3,000 transfection reagent and 0.3 pmol siRNA was added to each well. After 6 h of incubation, transfection media was replaced with growth media containing 0.5 µM NAP-6. Cells were incubated for a further 48 h prior to MTT analysis^23^.

### Xenobiotic response element assay

The activity of the AHR signalling pathway was measured using the Cignal Xenobiotic Response (XRE) Reporter Assay Kit from Qiagen according to the manufacturer’s instructions. Briefly, MDA-MB-468 cells were reverse transfected with the Cignal XRE Reporter (containing an AHR-responsive luciferase construct and a constitutively expressing Renilla luciferase) as well as positive and negative controls. After 20 h of transfection, the media was changed to assay media (DMEM with 0.5% FBS and 0.1 mM NEAA). After 24 h of transfection, cells were treated with NAP-6 (0.1 µM and 1.0 µM) for 6 h. The Dual-Glo Luciferase Assay System (Promega) was performed after 30 h of transfection using the GloMax Explorer Luminescence plate reader. The promoter activity was replicated twice and values are expressed as arbitrary units using Renilla reporter for internal normalization^23^.

### Gene expression analysis

For each cell population total RNA was extracted using the RNeasy Mini Kit (Qiagen) according to the manufacturer’s instructions. One microgram of RNA was reverse transcribed using the QuanitTect Reverse Transcription Kit (Qiagen) according to the manufacturer’s instructions. Rotor-Gene SYBR Green PCR Kit (Qiagen) was used to perform qPCR for AHR, CYP1A1, CYP1A2, CYP1B1, SULT1A1 and ARNT on a Rotor-Gene 3000 Thermo-Cycler Instrument using β_2_-microglobulin as a housekeeping gene (Qiagen). The primer sequences were purchased from Qiagen as follows: AHR (QT02422938), CYP1A1 (QT00012341), CYP1A2 (QT00000917), CYP1B1 (QT00209496), SULT1A1 (QT01665489), ARNT (QT00023177) AND β_2_M (QT00088935). HotStar Taq activation at 95 °C for 5 min, 40 cycles of denaturation (95 °C for 5 s), and annealing/extension (60 °C for 10 s). The comparative C_t_ value method was used for data analysis. Gene expression was examined in MDA-MB-468 cells following treatment with 1 µM NAP-6 for 1, 2, 4, 8, 12 and 24 h and in a broad panel of breast cell lines after 4 h treatment^23^.

### ER binding

Competition binding assays were performed by using an enzyme fragment complementation (EFC) method described in the HitHunter (Freemont, CA) EFC Estrogen Chemiluminescence Assay kit according to the manufacturer’s instructions. Briefly, competing ligands at final concentrations ranging from 25 pM to 2 µM were incubated with 5 nM recombinant ERα (Invitrogen) and 17β-estradiol-conjugated enzyme donor for 1.5 h. The enzyme acceptor was then added followed by the chemiluminescence substrate and incubated for 1 h. Relative luminescence was determined by using a GloMax Explorer plate reader (Promega). Sigmoidal standard curves were created by Excel^23^.

### Aromatase assay

Aromatase reactions were carried out as previously described^40^. Test chemicals were dissolved in DMSO and diluted 1:10 in Diluent 1 (0.1% BSA, 50 mM phosphate buffer (PB), pH7.2). Sample (10 µL) was added to a 96-well plate (on ice) followed by 50 µL of ice-cold R1 solution (0.1% BSA, 50 mM PB, pH 7.2, 3.3 mM NADP-2Na, BD Biosciences), 0.8 µM glucose-6-phosphate and 62.5 nM testosterone (Sigma). R2 solution (50 µL, 0.1% BSA, 50 mM PB, pH 7.2, 8.3 mM magnesium chloride and 1 U mL^−1^ glucose-6-phosphate dehydrogenase) was added to each test sample. 10 µL diluted P450arom (1 pg/mL, 0.1% BSA, 50 mM PB, pH 7.2, BD Biosciences) was added to a second 96-well plate on ice. 90 µL of sample reaction was transferred to the P450arom and incubated for 20 min at 37 °C. The reaction was terminated with 10 µL of 500 µM α-naphthoflavone. After completion of the P450aromatase reaction, 50 µL of sample was transferred to an ELISA plate. The amount of estradiol in each sample was determined using the Estradiol EIA kit (Cayman Chemical Company, Ann Arbor) according to the manufacturer’s instructions. Absorbance of each sample was proportional to the amount of bound estradiol tracer which was inversely proportional to the amount of estradiol^23^.

### Kinase inhibition

A dry sample of NAP-6 was sent to Reaction Biology Corp (PA, USA) and The International Centre for Kinase Profiling (The University of Dundee, UK) for kinase inhibition assays. Both organisations use the ^33^P ATP radioactive filter binding assay^41^. A stock solution of NAP-6 was prepared in DMSO and kinase inhibition assays were conducted in duplicate in the presence of a single concentration of NAP-6 (10 µM). Data represents percentage kinase enzyme activity, the lower the value the greater the enzyme inhibition^23^.

## Supplementary information

Supplementary Information
