# Supplementary material for: A novel naphthalimide that selectively targets breast cancer via the arylhydrocarbon receptor pathway
Source: Sci Rep. 2020 Aug 19;10:13978. doi: 10.1038/s41598-020-70597-8 (PMC7438328; doi:10.1038/s41598-020-70597-8)

Supplementary Information

Figure 1S. MDA-MB-468 cells were treated with NAP-6 (1.0 μM) for 0, 12 and 24 h, and examined for checkpoint activation (CHK2, pCHK2) and DNA damage (H2AXɣ) by Western blotting.

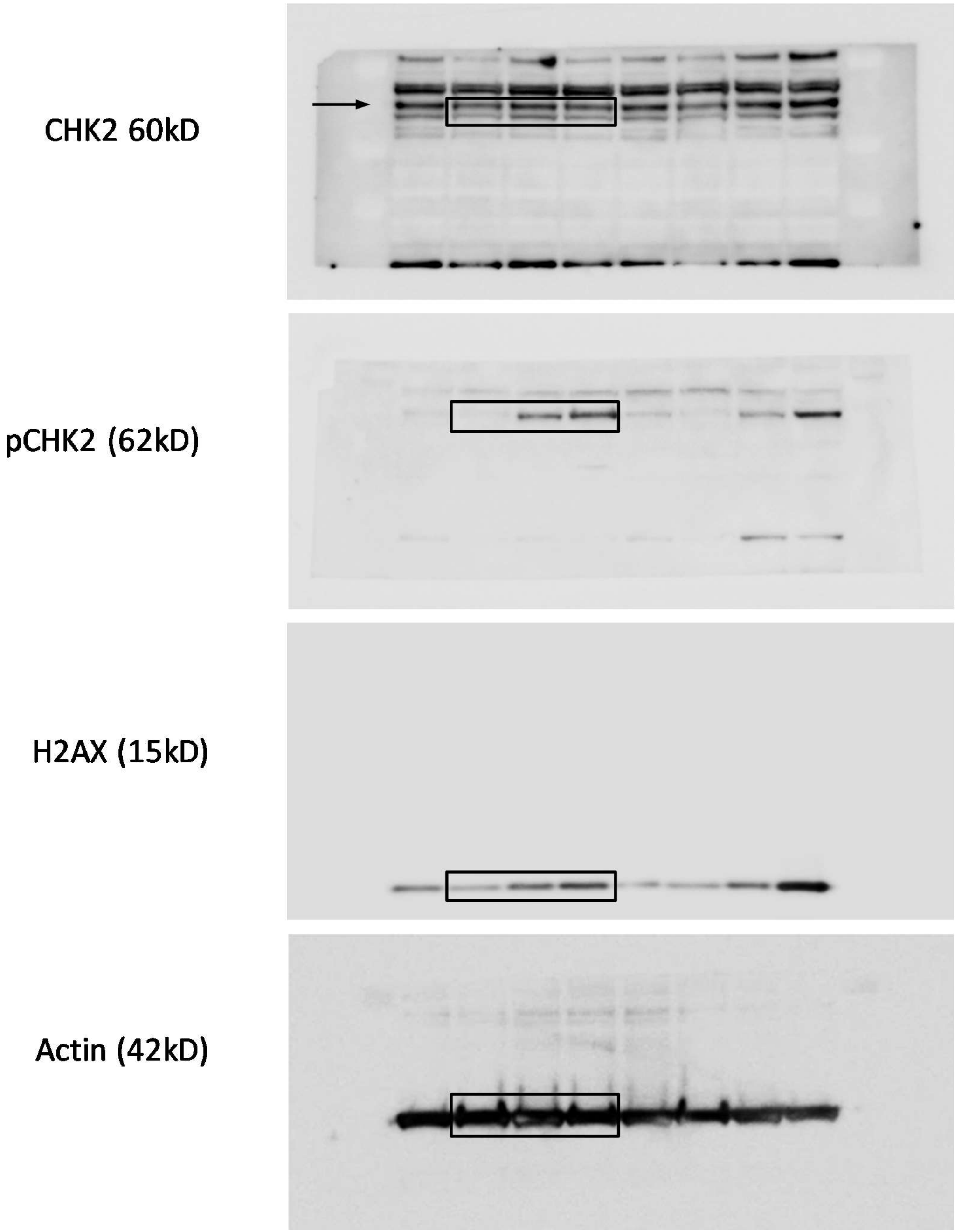

Figure 2S. Growth inhibition response (MTT assay) in MCF10A cells after 72 h of (a) NAP-6 (0.1 µM) in the presence and absence of the AHR antagonist CH223191 (5 µM) and (b) NAP-6 (1 µM) in the presence and absence of the CYP1 inhibitor α-naphthoflavone (αNF) (10 µM). Each data point is the mean ± SEM of three replicates.

a.

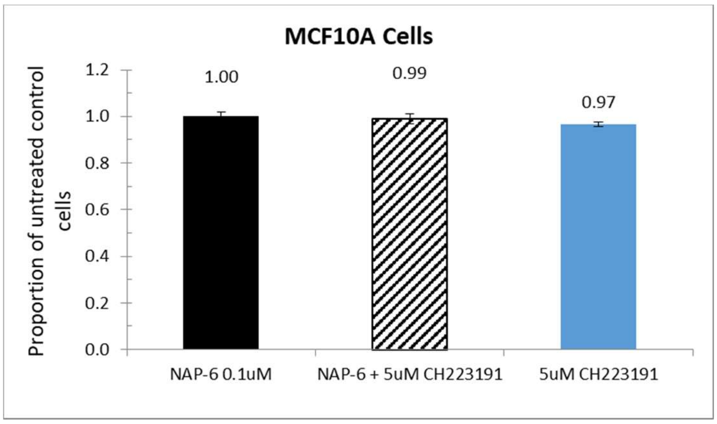

b.

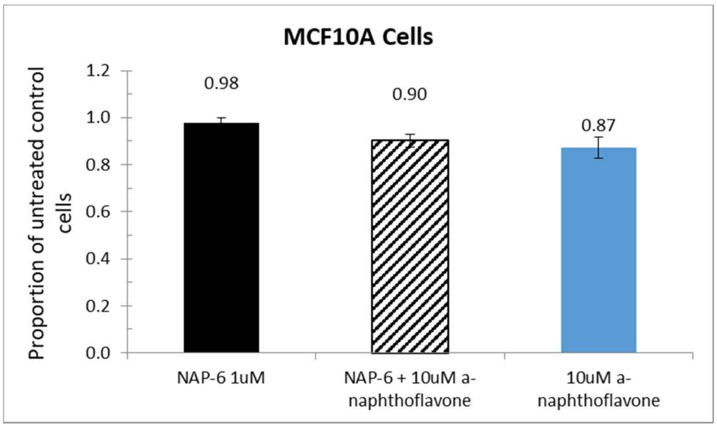

Supplement: Supplementary file 1 — Supplementary Information [file 41598_2020_70597_MOESM1_ESM.pdf]
